# Special Issue on Visual Sensors

**DOI:** 10.3390/s20030910

**Published:** 2020-02-08

**Authors:** Oscar Reinoso, Luis Payá

**Affiliations:** Department of Systems Engineering and Automation, Miguel Hernández University, 03202 Elche, Spain

## 1. Introduction

Visual sensors have characteristics that make them interesting as sources of information for any process or system. On the one hand, they are able to capture a very precise and high-resolution environmental information while occupying a small size and with a reduced price. On the other hand, they are able to capture a large quantity of information from the environment around them. These properties are the reason they have been employed for several decades for the resolution of multiple tasks. This high versatility in their fields of application makes them increasingly used as a source of information to solve a variety of diverse tasks.

Nowadays, a wide variety of visual systems can be found, from the classical monocular systems to omnidirectional, RGB-D, and more sophisticated 3D systems. Every configuration presents some specific characteristics that make them useful to solve different problems. Their range of applications is wide and varied. Among them, we can find robotics, industry, agriculture, quality control, visual inspection, surveillance, autonomous driving, and navigation aid systems.

Visual systems can be used to obtain relevant information from the environment, which can be processed to solve a specific problem. The aim of this Special Issue is to present some of the possibilities that vision systems offer, focusing on the different configurations that can be used and novel applications in any field.

In this Special Issue, 63 contributions were submitted and 36 of them were published (i.e., 57% acceptance rate). The published articles present a very adequate vision of how visual sensors are used in very different fields of application, from mapping for navigation of mobile robots to object recognition or scene reconstruction.

## 2. Contributions to the Special Issue on Visual Sensors

In the field of visual navigation of mobile robots, SLAM (Simultaneous Localization and Mapping), Visual odometry, etc., we find different alternatives that are presented in some of the papers of the Special Issue. Thus, in [1], an RGB-D SLAM algorithm is presented using the concept of orientation relevance taking into account the Manthattan Frame Estimation. Teng et al. [2] provided a method for aircraft pose estimation without relying on 3D models using two widely separated cameras to acquire the pose information. In [3], a new framework for online visual object tracking is proposed. A motion-aware strategy is employed to predict the possible region and scale of the target in the frame by utilizing the previously estimated 3D motion information. Wang et al. [4] provided an improved indoor visual SLAM method that uses point and line segment features extracted by stereo cameras, achieving robust results. In [5], an RGB-D sensor is employed. In this case, the purpose is to make a dense 3D semantic mapping of the environment by means of Pixel-Voxel network. Aladem et al. [6] proposed a low-overhead real-time ego-motion estimation (visual odometry) system based on either a stereo or RGB-D sensor. By means of the proposed algorithm, a local map is used, requiring significantly less memory and computational power. Nawaf et al. [7] provided the details of a visual odometry method adapted to the underwater context. They employed the captured stereo image to provide real-time navigation and a site coverage map, which is necessary to conduct a complete underwater survey. Valiente et al. [8] presented a visual information fusion approach for robust probability-oriented feature matching. This approach can be used in a more general SLAM procedure. This strategy permits obtaining relevant areas in the image reference system, from which probable matches could be detected.

Image retrieval aims at browsing, searching, and retrieving images from a large database of digital images. Proposing new descriptors of an image that define the characteristics of the image can be key in this regard. García-Olalla et al. [9] presented a new texture descriptor booster based on statistical information of the image. This descriptor is employed in texture-based classification images. Fareed et al. [10] proposed a framework for salient region detection that uses appearance-based and regression-based schemes to reduce the computational complexity and focusing on the salient parts of the image. In this sense, Feng et al. [11] proposed a texture descriptor for image retrieval designing a local parallel cross pattern in which the local binary pattern map is fused with the color map. In addition, Feng et al. [12] proposed a hybrid histogram descriptor used for image retrieval. The proposed descriptor comprises two histograms jointly: a perceptual uniform histogram and a motif co-occurrence histogram including the probability of a pair of motif patterns. Finally, García-Olalla et al. [13] proposed a method for textile based image retrieval for indoor environments based on describing the images with different channels (RGB, HSV, etc.) and using the combination of two different descriptors for the image.

Visual sensors can also be an important source of information to help and support for other tasks. Thus, in [14], a novel global point cloud descriptor is proposed for reliable object recognition and pose estimation, which can be applied to robot grasping operation. Martínez-Martin et al. [15] provided an approach based on depth cameras to robustly evaluate the manipulation success in robot object manipulation. The method proposed allows the robot to accurately detect the presence or absence of contact points between the robot manipulator and a held object. Xue et al. [16] presented a vision system capable of automatic 3D joint detection. The detection method is applied in a robotic seam tracking system for gas tungsten arc welding.

The calibration of vision systems plays a very important role in different applications where these types of sensors are used. Having a well-calibrated system will permit more robust results to be achieved in later stages. Zhang et al. [17] presented a simple calibration method for laser range finder systems needing only a calibration board. In [18], an alternative approach that uses gray-code patterns displayed on an LCD screen to determine camera parameters is provided. The proposed approach is 1.5 times more precise than using standard calibration with a checkerboard pattern. Finally, Choi et al. [19] proposed a method that automatically calibrates four cameras of an around view monitor system in a natural driving situation.

Object recognition is a task in which a vision system is almost always involved. During the past few years, many proposals have been made in this area including different methods that allow the recognition of the objects present in an image. In this way, Kapuscinski et al. [20] presented a method for hand shapes recognition based on skeletal data. It encodes the relative differences between vectors associated with the pointing direction of the particular fingers and the palm normal. Wang et al. [21] presented a new spatiotemporal action localization detector that consists of sequences of per-frame segmentation masks. This proposed detector can pinpoint the starting or ending frame of each action category in untrimmed videos. In [22], a system for automatically designing the field-of view of a camera, the illumination strength, and the parameters in a recognition algorithm is presented. Nguyen et al. [23] proposed a new presentation attack detection method for an iris recognition system using a near infrared light camera image. This method tries to avoid the effect that presentation attack images captured using high-quality printed images can cause in classic iris recognition systems. Fu et al. [24] presented an approach for pedestrian detection combining different methods previously proposed together with an efficient sliding window classification strategy. The detector achieves fast detecting speed at the same time as state-of-the-art accuracy. Wang et al. [25] proposed a model to resolve the 3D reconstruction problem for dynamic non-rigid objects with a single RGB-D sensor.

Over the past few years, the field of visual systems is shifting from classical statistical methods to deep learning methods. Video-based person detection and recognition is an important task with many problems and challenges such as lighting variation, occlusion, human appearance similarity, etc. In [26], a video-based person re-identification method with hybrid deep appearance-temporal features is proposed. Another application using deep learning methods was presented by Arsalan et al. [27]. The authors proposed a densely connected fully convolutional network, which can determine the true iris boundary even with inferior-quality images by using better information gradient flow between the dense blocks. Liu et al. [28] proposed a method to improve the performance of the star sensor under dynamic conditions based on the ensemble back-propagation neural network.

Scene reconstruction is a key task necessary to accomplish more complex problems such as mobile robot navigation. Xia et al. [29] presented a visual inertial odometry as a solution to the robot navigation system. Cheng et al. [30] presented a high-accuracy method for globally consistent surface reconstruction using a single fringe projection profilometry sensor. Lane marking detection and localization are crucial for autonomous driving and lane-based pavement surveys. In [31], a novel methodology is presented for automated lane marking identification and reconstruction. In addition, a case study is given to validate the proposed methodology. Finally, Zhang et al. [32] proposed an improved method for UAV image seamline searching. The experimental results show that the proposed method can effectively solve the problems of ghosting and seams in the panoramic UAV images.

Finally, one of the most widely discussed topics about vision systems is to establish visual measurements. Some of the papers of the Special Issue revolve around this problem. In [33], the authors presented an improved rotation-angle measurement method based on geometric moments that is suitable for automatic sorting systems. In [34], a stereo vision system is employed for measuring the ram speed of steam hammers. The system tries to decrease the influence of strong vibration. The accuracy and effectiveness of the method was experimentally verified. Li et al. [35] proposed a pose estimation method for sweet pepper detachment. The acquired point cloud is separated into candidate planes that are separately evaluated using a scoring strategy. Yang et al. [36] presented a comparative analysis of digital image correlation based stereo 3D shape measurements.
